# Active learning in undergraduate classroom dental education- a scoping review

**DOI:** 10.1371/journal.pone.0293206

**Published:** 2023-10-26

**Authors:** Arnaldo Perez, Jacqueline Green, Mohammad Moharrami, Silvia Gianoni-Capenakas, Maryam Kebbe, Seema Ganatra, Geoff Ball, Nazlee Sharmin

**Affiliations:** 1 School of Dentistry, Faculty of Medicine & Dentistry, University of Alberta, Edmonton, Alberta, Canada; 2 Faculty of Dentistry, University of Toronto, Toronto, Ontario, Canada; 3 Faculty of Kinesiology, University of New Brunswick, Fredericton, New Brunswick, Canada; 4 Department of Pediatrics, Faculty of Medicine & Dentistry, University of Alberta, Edmonton, Alberta, Canada; University of Eastern Finland: Ita-Suomen yliopisto, FINLAND

## Abstract

**Introduction:**

Previous reviews on active learning in dental education have not comprehensibly summarized the research activity on this topic as they have largely focused on specific active learning strategies. This scoping review aimed to map the breadth and depth of the research activity on active learning strategies in undergraduate classroom dental education.

**Methods:**

The review was guided by Arksey & O’Malley’s multi-step framework and followed the PRISMA Extension Scoping Reviews guidelines. MEDLINE, ERIC, EMBASE, and Scopus databases were searched from January 2005 to October 2022. Peer-reviewed, primary research articles published in English were selected. Reference lists of relevant studies were verified to improve the search. Two trained researchers independently screened titles, abstracts, and full-texts articles for eligibility and extracted the relevant data.

**Results:**

In total, 93 studies were included in the review. All studies performed outcome evaluations, including reaction evaluation alone (n = 32; 34.4%), learning evaluation alone (n = 19; 20.4%), and reaction and learning evaluations combined (n = 42; 45.1%). Most studies used quantitative approaches (n = 85; 91.3%), performed post-intervention evaluations (n = 70; 75.3%), and measured student satisfaction (n = 73; 78.5%) and knowledge acquisition (n = 61; 65.6%) using direct and indirect (self-report) measures. Only 4 studies (4.3%) reported faculty data in addition to student data. Flipped learning, group discussion, problem-based learning, and team-based learning were the active learning strategies most frequently evaluated (≥6 studies). Overall, most studies found that active learning improved satisfaction and knowledge acquisition and was superior to traditional lectures based on direct and indirect outcome measures.

**Conclusion:**

Active learning has the potential to enhance student learning in undergraduate classroom dental education; however, robust process and outcome evaluation designs are needed to demonstrate its effectiveness in this educational context. Further research is warranted to evaluate the impact of active learning strategies on skill development and behavioral change in order to support the competency-based approach in dental education.

## Introduction

Active learning (AL) has been broadly defined as a type of learning that demands active gathering, processing, and application of information rather than passive assimilation of knowledge [1]. This form of learning is well aligned with principles of adult learning, including self-direction, purposefulness, experience-based, ownership, problem orientation, mentorship, and intrinsic motivation [2]. Because students regularly enroll in dental programs as young adults after completing an undergraduate degree, active learning has been encouraged in dental education to help students gain knowledge and develop basic and advanced dental, cognitive, and social skills [3]. Active learning, along with curricular integration, early exposure to clinical care, and evidence-based teaching and assessment are important reforms introduced in dental education to ensure that students develop the competencies they need to become entry-level general dentists in the 21st century [4].

Numerous teaching strategies have been developed to promote active learning across health professions education, including problem-based learning, case-based learning, flipped learning, team-based learning, and group discussion. Research suggests that students and instructors positively value active learning [5, 6]; however, inconclusive evidence exists on the actual impact of active learning on knowledge acquisition, skill development, and attitudinal change in health sciences education [7, 8].

Many studies have been conducted on active learning in dental education, especially in the last two decades. Some primary and review studies have found that active learning is well received by students and instructors and may be more effective than traditional lecture-based teaching in dental education [9, 10]. However, review studies, in particular, have fallen short of providing a comprehensive overview of the existing literature on active learning in dental education [9, 11, 12]. For example, they have largely focused on the outcomes of a few active learning strategies (e.g., problem-based learning, flipped learning) providing limited data on their implementation and evaluation designs. These review studies have also failed to differentiate the scope, range, and nature of the research activity on active learning in different learning environments, including classroom dental education. This learning environment has unique characteristics and is of particular importance because it provides the foundational knowledge that students are expected to apply in laboratory and clinical settings.

Our scoping review aimed to map the breadth and depth of the research activity on active learning strategies in undergraduate classroom dental education from January 2005 to October 2022. Mapping this extensive body of literature is important to inform future research directions on active learning in dental education.

## Methods

The scoping review framework developed by Arksey & O’Malley (2005) guided the study design, which includes the following stages: (1) formulating research questions, (2) identifying potentially relevant studies, (3) selecting relevant studies, (4) charting the data, and (5) collating, summarizing, and reporting results [13]. Unlike systematic reviews that typically synthesize the existing evidence on relationships between exposure and outcome variables, scoping reviews are well suited to map the breadth and depth of the research activity on complex topics and identify gaps in the relevant literature [13]. Our review report followed the guidelines of PRISMA Extension for Scoping Reviews [14].

### Stage 1: Formulating research questions

Our scoping review sought to answer the following questions:

- What are the characteristics of the studies conducted on active learning in classroom dental education in the study period?
- How were active learning strategies evaluated?
- What were the main results of the studies conducted?

### Stage 2: Identifying potentially relevant studies

Four databases (MEDLINE, ERIC, EMBASE, and Scopus) were searched from January 2005 to October 2022. A preliminary search suggested that most studies on the study topic were published in the last two decades and the quality of the reports produced had substantially improved in the same study period. The search strategy for MEDLINE was developed by two authors (JG and AP) in consultation with a librarian at the University of Alberta. This strategy was then adapted for each database included in the review. Search terms used in each database are shown in Table 1. Reference lists of included studies and articles selected in previous reviews on specific active learning strategies were verified to enhance the search and test its sensitivity.

**Table 1:** Detail of search terms and search results.

Database	Search Terms	Search Results (Number of Papers)	Year
MedLine	[active learn. OR problem based learning.mp. or exp Problem-Based Learning/ OR case based learning.mp. OR Group adj2 discuss*).mp. OR (small adj2 group*).mp. OR (small adj2 group*).mp. OR (peer adj2 teach*).mp. OR (critical adj2 think*).mp. OR (role adj2 play*).mp. OR team based learning.mp. OR (peer adj2 learn*).mp. OR (flipped adj2 class*).mp. OR (flipped adj2 learn*).mp. OR (blended adj2 learn*).mp.] AND [class.mp. OR class*.mp. OR classes.mp. OR preclinical.mp. OR non-clinical.mp. OR in-class.mp. OR course.mp. OR courses.mp.] AND [exp Students, Dental/ OR exp Education, Dental/ OR (dental adj2 learn*).mp. OR ((dental or dentist*) adj2 (educat* or learn* or student* or teach* or instruct* or curricul*)).mp. OR exp Schools, Dental/]	422	2005–2022
ERIC	[exp Active Learning/ or active learn*.mp. OR Case based learning.mp. or exp "Case Method (Teaching Technique)"/ OR case-based learning.mp. OR problem based learning.mp. or exp Problem Based Learning/ OR problem-based learning.mp. OR (think* adj1 pair* adj1 share*).mp. OR (peer* adj2 learn*).mp. OR critical adj2 think*).mp. OR exp Critical Thinking/ OR (role adj2 play).mp. OR exp Classrooms/ or class*.mp. OR discuss*.mp. or exp Discussion Groups/ or exp Discussion/ or exp Group Discussion/ OR reflection.mp. or exp Reflection/ OR teaching methods.mp. or exp Teaching Methods/] AND [((dental or dentist*) adj2 (educat* or learn* or student*)).mp. OR undergraduate dent*.mp. OR dental schools.mp. or exp Dental Schools/ OR exp Dentistry/ OR dental college.mp.]	132	2005–2022
Scopus	[active learn* OR Problem based Learn* OR Case based learn* OR Group discuss* OR *think pair share* OR Peer learn* OR "peer teach* OR critical think* OR Role play* OR flipped learn* OR Flipped Class* OR blended learn*] AND [Class* OR *preclinical* OR *non-clinical* OR *in-class* OR course*] AND [*dental school* OR *dentistry* OR dental learn* OR dental educat* OR dental student* OR dental teach* OR dental instruct* OR dental curricul*]	442	2005–2022
EMBASE	[active learn*.mp. OR problem based learning.mp. or exp Problem Based Learning/ OR exp problem based learning/ OR case-based learning.mp. OR (Group adj2 discuss*).mp. OR (small adj2 group*).mp. OR (think* adj1 pair* adj1 share*).mp. OR (peer adj2 learn*).mp. OR (peer adj2 teach*).mp. OR (critical adj2 think*).mp. OR (role adj2 play*).mp. OR team based learning.mp. OR (peer adj2 learn*).mp. OR (flipped adj2 class*).mp. OR (flipped adj2 learn*).mp. OR teaching methods.mp. or exp teaching/ OR (blended adj2 learn*).mp.] AND class*.mp. OR preclinical.mp. OR non-clinical.mp. OR in-class.mp. OR course*.mp.] AND [exp dental student/ OR exp dental education/ OR (dental adj2 learn*).mp. OR ((dental or dentist*) adj2 (educat* or learn* or student* or teach* or instruct* or curricul*)).mp. OR dental school.mp.]	1200	2005–2022

### Stage 3: Selecting relevant studies

Inclusion and exclusion criteria were based on the research questions and refined during the screening process. Primary studies published in English were included if they met the following criteria: (i) focused on undergraduate dental education in classroom settings, (ii) used at least one active learning strategy, (iii) involved dental students, and (iv) reported dental student data when students from other programs (e.g., medical students) were involved in the study. Studies were excluded if they were published in a language other than English, reported active learning in clinical or laboratory settings or at program level, and were not available as full-text articles. Review studies and perspective articles were also excluded. No restrictions were set on research methods. All references were exported to Zotero and duplicates were removed by JG. The remaining papers were then exported to Rayyan. A training session was held to ensure understanding of inclusion and exclusion criteria and consistency in their application. Two researchers (JG and SGC) independently screened for titles and abstracts and three researchers independently reviewed the full texts of articles selected in the first phase of screening (JG, SGC, MM). Consensus was obtained by discussion or consulting a fourth reviewer (AP).

### Stage 4: Charting the data

A piloted, literature-informed data collection form was used to extract data on publication (year of publication, country of publication), study characteristics (research inquiry, research methodology, means of data collection), participant characteristics (type of student, sample size), intervention (content area, active learning strategy, comparator, and length of the exposure), evaluation (type of evaluation, level of evaluation, evaluation design, and outcome of interest) and main findings. Data extraction was completed independently by two trained researchers (JG and MM) and the completed data extraction forms were compared. Consensus was obtained by discussion or consulting a third reviewer (AP). Authors of studies that did not report key aspects included in the data extraction form were contacted to provide that information. Missing information was then categorized as “not reported.”

### Stage 5: Collating, summarizing, and reporting results

Descriptive statistics were used to summarize quantifiable data using previously developed or data-driven classifications. Evaluation data such as level, outcomes (directly and indirectly measured), and results were summarized according to Kirkpatrick’s Model (1998) [15]. This model suggests four levels of outcome evaluation, including *reaction* (satisfaction and perceived outcomes), *learning* (direct measures of outcomes such knowledge, skills, and attitudes), *behavior* (behavioral changes resulting from the intervention), and *results* (organizational changes resulting from the intervention).^15^ This model is widely used to describe evaluations of educational interventions in a variety of contexts. Papers reporting more than one outcome level and active learning strategy were classified separately to calculate the number of evaluations per level and active learning strategy, respectively.

## Results

**Fig 1:**
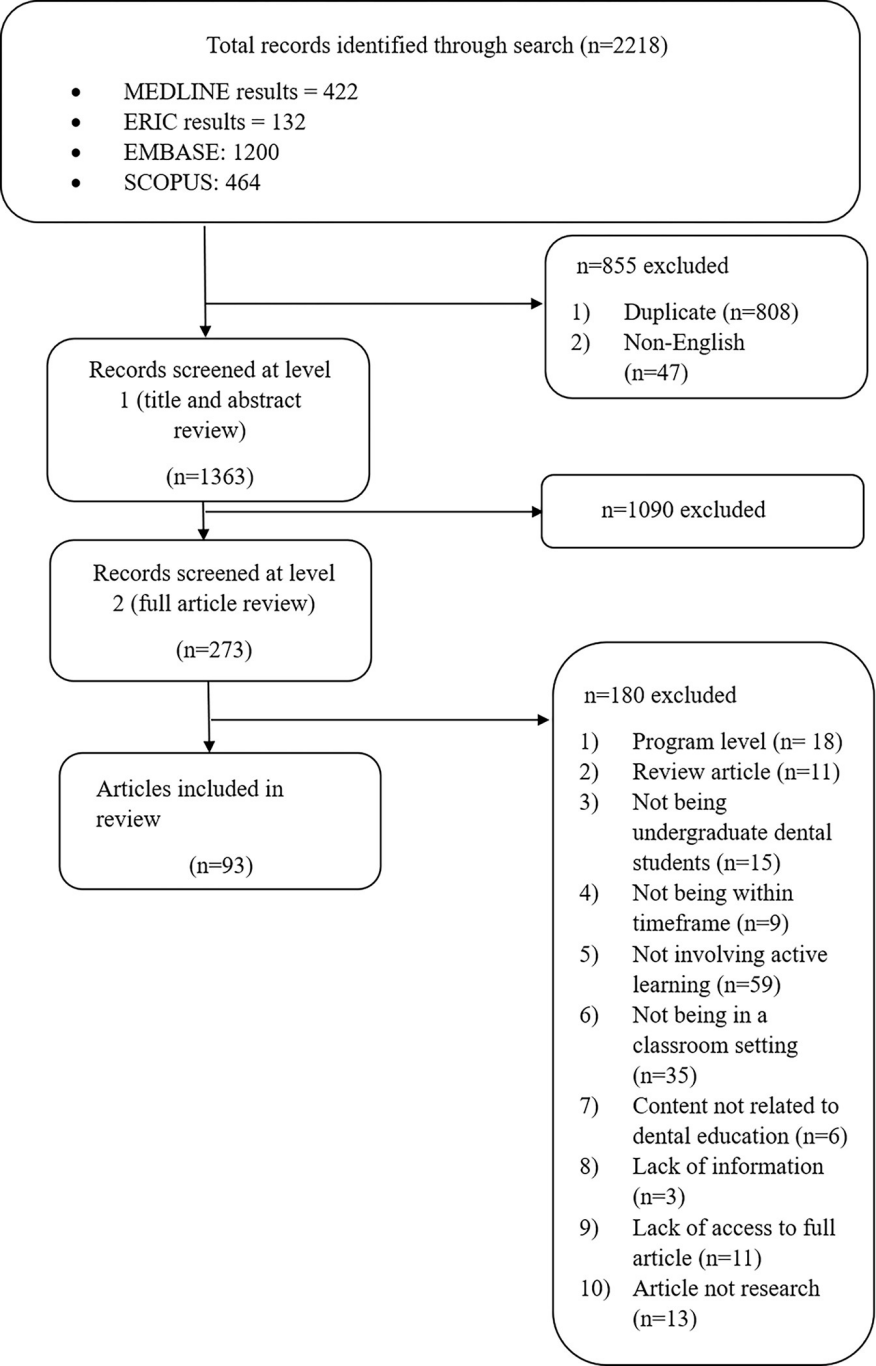
Flow diagram of the study selection process.

Searches in EMBASE (n = 1200), MEDLINE (n = 422), Scopus (n = 464), and ERIC (n = 132) databases generated 2,218 records. Duplicates (n = 808) and articles not published in English (n = 47) were removed. The screening of titles and abstracts yielded 273 potentially eligible articles and the screening of full texts identified 93 eligible articles, which were included in this review (Fig 1). No additional articles were identified through checking the reference lists of eligible studies and studies included in previous. A total of 10,473 students and 199 faculty were involved in the selected studies. Students involved were from dentistry (n = 10,297; 98.3%), medicine (n = 126; 1.2%), and dental hygiene (n = 50; 0.5%).

### Characteristics of reviewed studies

As shown in Table 2, selected studies originated from different geographical areas, including Asia (n = 46; 49.4%), North America (n = 29; 31.1%), Europe (n = 10; 10.7%), South America (n = 6; 6.4%), Australia (1) and Africa (1). Twenty-eight of the studies produced in North America were conducted in the United States and 1 in Canada. Thirty-one studies were published between 2005 and 2014 and 62 between 2015 and 2022. Nine studies (9.6%) did not indicate the content area. Most studies reported active learning in clinical (n = 54; 58%) and basic (n = 25; 26.8%) sciences, and only 5 (5.3%) in behavioral and social sciences.

**Table 2:** Summary of characteristics of reviewed studies.

Authors, year	Country	Inquiry	Study Design	Content Area	Active Learning Strategies	Comparator (if any)	Level of Evaluation
Mitchell & Brackett, 2017 [35]	USA	Quantitative	Not reported	Basic sciences	Flipped learning with TBL*	Traditional lecture	Reaction
Omar, 2017 [36]	Saudi Arabia	Quantitative	Not reported	Clinical sciences	Group discussions	N/A	Reaction
Gali et al., 2015 [37]	India	Quantitative	RCT**	Basic sciences	Group discussions	Traditional lecture	Reaction and Learning
Ihm et al., 2017 [38]	Korea	Quantitative	Not reported	Basic sciences	Flipped learning	Traditional lecture	Reaction
Kim et al., 2018 [39]	Korea	Quantitative	Not reported	Basic sciences	Flipped learning	Traditional lecture	Reaction and Learning
Luchi et al., 2017 [40]	Brazil	Quantitative	Not reported	Basic sciences	Game	Traditional lecture	Reaction and Learning
Almajed et al., 2016 [41]	Australia	Qualitative	Not reported	Not reported	Group discussion	Traditional lecture	Reaction
Ha-Ngoc & Park, 2015 [42]	USA	Quantitative	Not reported	Clinical sciences	Peer teaching	Traditional lecture	Reaction
Park et al., 2014 [43]	USA	Quantitative	Not reported	Clinical sciences	TBL***	Individual learning	Learning
Miller et al., 2013 [44]	USA	Quantitative	Not reported	Basic sciences	Think-pair-share	Traditional lecture	Reaction and Learning
Khan, 2011 [45]	South Africa	Quantitative	Not reported	Clinical sciences	Group discussion	Active learning activities	Reaction
Kieser et al., 2008 [46]	New Zealand	Quantitative	Not reported	Clinical sciences	PBL	PBL	Reaction
Reich et al., 2007 [47]	Germany	Quantitative	Not reported	Clinical sciences	PBL	Traditional lecture	Reaction and Learning
Qutieshat et al., 2020 [48]	Jordan	Quantitative	Not reported	Clinical sciences	Flipped learning	Traditional lecture	Reaction and Learning
Ashwini et al., 2019 [49]	India	Quantitative	Not reported	Behavioral Sciences	Flipped learning	Traditional lecture	Reaction
Kohli et al., 2019 [50]	Malaysia	Quantitative	Cohort study	Clinical sciences	Flipped learning	Traditional lecture	Reaction and Learning
Tricio et al., 2019 [51]	Columbia	Mixed method	Not reported	Clinical sciences	Fishbowl	Traditional lecture	Reaction and Learning
Tauber et al., 2019 [52]	Czech Republic	Quantitative	Not reported	Basic sciences	Group discussion	Traditional lecture	Reaction and Learning
Himida et al., 2019 [53]	Scotland	Mixed method	Not reported	Behavioral sciences	Forum theatre	Traditional lecture	Reaction
Slaven et al., 2019 [54]	USA	Quantitative	Not reported	Clinical sciences	Flipped learning	Traditional lectures	Reaction and Learning
Park et al., 2019 [55]	USA	Quantitative	Not reported	Clinical sciences	TBL	Individual learning	Reaction and Learning
Yang et al., 2019 [56]	China	Quantitative	Not reported	Basic sciences	Group discussion	Traditional lectures	Reaction and Learning
Veeraiyan et al., 2019a [57]	India	Quantitative	Not reported	Basic sciences	TBL	Traditional lectures	Reaction and Learning
Veeraiyan et al., 2019b [58]	India	Quantitative	Retrospective	Clinical sciences	Flipped learning	Traditional lectures	Learning
Veeraiyan et al., 2019c [59]	India	Quantitative	Prospective	Clinical sciences	Flipped learning	Traditional lectures	Learning
Veeraiyan et al., 2019d [60]	India	Quantitative	Not reported	Clinical sciences	Flipped learning	Traditional lectures	Learning
Al-Madi et al., 2018 [61]	Saudi Arabia	Quantitative	Cross-sectional	Basic sciences	PBL	Traditional lectures	Reaction and Learning
Chutinan et al., 2018 [62]	USA	Mixed method	Not reported	Basic sciences	Flipped learning	Traditional lectures	Reaction and Learning
Jones, 2019 [63]	USA	Mixed method	Not reported	Clinical sciences	Group discussion	Traditional lectures	Reaction and Learning
Xiao et al., 2018 [64]	USA	Quantitative	Comparative	Basic sciences	Flipped learning	Traditional lectures	Reaction and Learning
Varthis & Anderson, 2018 [65]	USA	Quantitative	Not reported	Basic sciences	Blended learning	Traditional lectures	Reaction
Islam et al., 2018 [66]	Malaysia	Quantitative	Case control	Clinical sciences	Flipped learning	Traditional lectures	Reaction and Learning
Lee & Kim, 2018 [67]	USA	Quantitative	Not reported	Clinical sciences	Flipped learning	Traditional lectures	Reaction and Learning
Costa-Silva et al., 2018 [68]	Brazil	Quantitative	Not reported	Basic sciences	Group discussion	Traditional lectures	Learning
AbdelSalam et al., 2017 [69]	Saudi Arabia	Quantitative	Not reported	Basic sciences	Peer teaching	Traditional lectures	Learning
Bai et al., 2017 [70]	China	Mixed method	RCT	Clinical sciences	PBL	Traditional lectures	Reaction and Learning
Nishigawa et al., 2017a [71]	Japan	Quantitative	Cohort	Clinical sciences	TBL	Traditional lectures	Learning
Gadbury-Amyot et al., 2017 [72]	USA	Quantitative	Not reported	Clinical sciences	Flipped learning	Traditional lectures	Reaction
Sagsoz et al., 2017 [73]	Turkey	Quantitative	Pre- and post- test	Clinical sciences	Jigsaw method	Traditional lectures	Learning
Nishigawa et al., 2017b [74]	Japan	Quantitative	Not reported	Clinical sciences	Flipped learning	TBL	Learning
Samuelson et al., 2017 [75]	USA	Quantitative	Crossover	Clinical sciences	Group discussion	Traditional lectures	Reaction and Learning
Eachempati et al., 2016 [76]	Malaysia	Qualitative	Cross-sectional	Clinical sciences	Blended learning with group learning	Traditional lectures	Reaction
Cardozo et al., 2016 [77]	Brazil	Quantitative	Not reported	Basic sciences	Game	Traditional lectures	Learning
Bohaty et al., 2016 [78]	USA	Quantitative	Not reported	Clinical sciences	Flipped learning	Traditional lectures	Reaction
Echeto et al., 2015 [79]	USA	Quantitative	Not reported	Clinical sciences	TBL	Traditional lectures	Learning
Park & Howell, 2015 [80]	USA	Quantitative	Not reported	Basic Sciences	Flipped learning	Traditional lectures	Reaction
Takeuchi et al., 2015 [81]	Japan	Quantitative	Not reported	Clinical sciences	TBL	Traditional lectures	Reaction and Learning
Ilgüy et al., 2014 [82]	Turkey	Quantitative	Not reported	Clinical sciences	Group discussion	Traditional lectures	Learning
Guven et al., 2014 [83]	Turkey	Quantitative	Not reported	Basic sciences	PBL	Traditional lectures	Reaction and Learning
Du et al., 2013 [84]	China	Quantitative	Not reported	Clinical sciences	Group discussion	Traditional lectures	Reaction and Learning
Haj-Ali & Al Quran, 2013 [85]	United Arab Emirates	Quantitative	Not reported	Clinical sciences	TBL	Traditional lectures	Reaction and Learning
Ratzmann et al., 2013 [86]	Germany	Quantitative	Not reported	Clinical sciences	PBL	Traditional lectures	Reaction
McKenzie, 2013 [87]	USA	Quantitative	Pre-and post-test	Clinical sciences	Group discussion	Traditional lectures	Reaction
Kumar & Gadbury-Amyot, 2012 [88]	USA	Quantitative	Not reported	Clinical sciences	TBL	Traditional lectures	Reaction and Learning
Alcota et al., 2011 [89]	Chile	Quantitative	Not reported	Clinical sciences	PBL with debate and group discussion	Traditional lectures	Reaction and Learning
Romito & Eckert, 2011 [90]	USA	Quantitative	Not reported	Basic sciences	PBL	Traditional lecture	Learning
Obrez et al., 2011 [91]	USA	Quantitative	Not reported	Basic sciences	Group discussion	Traditional lecture	Reaction and Learning
Dantas et al., 2010 [92]	Brazil	Quantitative	Not reported	Clinical sciences	Group discussion	Text reading	Learning
Grady et al., 2009 [93]	UK	Quantitative	Not reported	Clinical sciences	Group discussion	Traditional lecture	Reaction
Moreno-López et al., 2009 [94]	Italy	Quantitative	Not reported	Clinical sciences	PBL	Traditional lecture	Learning and Reaction
Pileggi & O’Neill, 2008 [95]	USA	Quantitative	Not reported	Clinical sciences	TBL	Traditional lecture	Reaction and Learning
Park et al., 2007 [96]	USA	Quantitative	Retrospective	Clinical sciences	PBL with tutor expertise	PBL without tutor expertise	Learning and Reaction
Rich et al., 2005 [97]	USA	Quantitative	Not reported	Clinical sciences	PBL	Traditional lecture	Reaction
Croft et al., 2005 [98]	UK	Quantitative	Not reported	Behavioral sciences	Role Play	Traditional lecture	Reaction
Deepak et al., 2019 [58]	India	Quantitative	Prospective	Clinical sciences	Flipped learning	Traditional lecture	Learning
Qutieshat et al., 2018 [99]	Jordan	Quantitative	Not reported	Clinical sciences	Debate	Reply Speech	Reaction
Paul et al., 2019 [100]	Malaysia	Quantitative	Cross-sectional	Clinical sciences	Blended learning	Traditional lecture	Reaction and Learning
Youssef et al., 2012 [101]	Egypt	Quantitative	Not reported	Basic sciences	Group discussion	Traditional lecture	Reaction
Al Kawas & Hamdy, 2017 [102]	United Arab Emirates	Mixed method	Not reported	Not reported	TBL	Traditional lecture	Reaction
Nishigawa et al., 2017c [74]	Japan	Quantitative	Not reported	Clinical sciences	TBL and flipped learning	Flipped learning	Reaction and Learning
Khan et al., 2012 [103]	Malaysia	Quantitative	Not reported	Basic sciences	Debate	Traditional lecture	Reaction
Katsuragi, 2005 [104]	Japan	Quantitative	Not reported	Basic sciences	PBL	Traditional lecture	Reaction and Learning
Zhang et al., 2012 [105]	China	Quantitative	Not reported	Clinical sciences	PBL	Traditional lectures	Reaction and Learning
Zain-Alabdeen, 2017 [106]	Saudi Arabia	Quantitative	Not reported	Clinical sciences	Flipped learning	Traditional lectures	Reaction
Elledge et al., 2018 [107]	UK	Quantitative	Not reported	Clinical sciences	Flipped learning	Traditional lectures	Reaction and Learning
Richards & Inglehart, 2006 [108]	USA	Quantitative	Not reported	Clinical sciences	Group discussion	Traditional lectures	Reaction
Tack & Plasschaert, 2006 [109]	Netherlands	Quantitative	Not reported	Clinical sciences	PBL	Traditional lectures	Reaction and Learning
Markose et al., 2018 [110]	India	Quantitative	Comparative	Behavioral sciences	PBL	Traditional lectures	Reaction and Learning
Ahmadian et al., 2017 [111]	Iran	Quantitative	Interventional	Behavioral sciences	PBL	Role play	Reaction
Metz et al., 2015 [112]	USA	Quantitative	Not reported	Clinical sciences	Group discussion	Traditional lectures	Reaction and Learning
Shigli et al., 2017 [113]	India	Quantitative	Experiment	Clinical sciences	Group discussion	Traditional lectures	Reaction
Roopa et al., 2013 [114]	India	Quantitative	Not reported	Basic sciences	Peer teaching	Traditional lectures	Reaction
Rimal et al., 2015 [115]	Nepal	Quantitative	Not reported	Basic sciences	PBL	Traditional lectures	Reaction
Ihm et al., 2017 [116]	Republic of Korea	Quantitative	Not reported	Not reported	PBL	Traditional lectures	Learning
Chandelkar & Kulkarni, 2014 [117]	India	Quantitative	Not reported	Basic sciences	Peer teaching	Traditional lectures	Reaction and Learning
Huynh et al., 2022 [118]	USA	Quantitative	Not reported	Clinical sciences	Blended Learning	Traditional lectures	Reaction
Özcan, 2022 [119]	USA	Quantitative	Not reported	Clinical sciences	Flipped Learning	Traditional lectures	Learning and Reaction
Gallardo et al., 2022 [120]	Spain	Quantitative	Pre- and post-test	Clinical sciences	Flipped Learning	Traditional lectures	Reaction
Alharbi et al., 2022 [121]	Saudi Arabia	Quantitative	Pre- and post-test	Not reported	Flipped Learning	Traditional lectures	Learning and Reaction
Zhou et al., 2022 [122]	China	Quantitative	Pre- and post-test	Clinical sciences	Flipped Learning	Traditional lectures	Reaction
Xiao et al., 2021 [123]	USA	Quantitative	Pre- and post-test	Basic science	Flipped Learning	Traditional lectures	Learning
Veeraiyan et al., 2022 [124]	India	Quantitative	Not reported	Not reported	Multiple active learning strategies	NA	Learning
Ganatra et al., 2021 [125]	Canada	Mixed method	Not reported	Clinical sciences	Think pair share	NA	Reaction

*TBL: Team-based learning

**RCT: Randomized control trial

***PBL: Problem-based learning

Methodologically, most studies (n = 85; 91.3%) were quantitative in nature. Only a few used qualitative (n = 2; 2.1%) and mixed-method (n = 6; 6.4%) approaches. Most studies (n = 67; 72%) did not explicitly report the methodology used and some (n = 8; 8.6%) reported features of the methodology employed (e.g., prospective, comparative). Reported quantitative methods (n = 26; 27.9%) included pre- and post-tests (n = 6), randomized controlled trials (n = 4), cross-sectional studies (n = 3), cohort studies (n = 2), qualitative description (n = 1), case-control studies (n = 1), and experiments without randomization (n = 1). Two reported randomized controlled trials did not describe sequence generation, none reported allocation concealment details, and only 1 reported blinding of outcome assessors. Most common means of data collection included surveys (n = 74; 79.5%) and test scores (n = 59; 63.4%) alone or combined.

### Evaluation types and designs

All studies performed outcome evaluations. No process evaluations were reported alone or combined with outcome evaluations. Outcomes evaluated included satisfaction (n = 73), knowledge acquisition (n = 61), skill development (e.g., clinical, problem-solving, communication skills) (n = 3), and engagement (n = 2). Studies performed post-intervention (n = 70; 75.2%), pre-post-intervention (n = 18; 19.3%), and during-post-intervention (n = 5; 5.3%) evaluations.

Of all the evaluations performed (n = 93), post-intervention evaluations (n = 70) included a single group exposed to one condition (n = 23; 24.7%) or two compared conditions (n = 9; 9.6%), two compared groups exposed to two conditions including (n = 10; 10.7%) and not including (n = 21; 22.5%) randomization, and two or more non-compared groups exposed to one condition, including one-time (n = 6; 6.4%) or two-time (n = 1; 1.07%) evaluation points. In the one-time evaluation point, the outcome variables of interest were evaluated after the intervention, whereas in the two-time evaluation points, the outcome variables of interest were evaluated after the intervention by asking participants to assess those variables before and after the intervention. In both cases, the evaluation data of the study groups were aggregated. Pre-and-post intervention evaluations (n = 18), included a single group exposed to one condition (n = 4; 4.3%) or two compared conditions (n = 1; 1.07%), two compared groups exposed to two conditions including (n = 8; 8.6%), and not including (n = 4; 4.3%) randomization, and two or more non-compared groups exposed to one condition with one-time evaluation point (n = 1; 1.07%). During-post-intervention evaluations (n = 5), included a single group exposed to one condition (n = 1; 1.07%) or two compared conditions (n = 1; 1.07%) and two compared groups exposed to two conditions including (n = 1; 1.07%) and not including (n = 2; 2.1%) randomization.

### Evaluated active learning strategies

Studies evaluated several active learning strategies. Strategies frequently (more than 10 studies) and fairly (between 6 and 10 studies) evaluated included flipped learning, group discussion, problem-based learning (PBL), and team-based learning (TBL). Blended learning, peer teaching, debate, and role play were occasionally evaluated (between 3 to 5 studies). Strategies seldom evaluated (1 or 2 studies) included games, think-pair-share, and others such as fishbowl and Jigsaw. All outcome evaluations were performed at reaction and learning levels as the present review focused on classroom dental education. Thirty-two studies (34.4%) performed reaction evaluations alone, 19 (20.4%) learning evaluations alone, and 42 (45.1%) reaction and learning evaluations combined. Only 4 studies (4.3%) reported faculty data in addition to student data. The lengths of the exposures to active learning ranged from one hour to three years.

### Reaction-level evaluations, including self-reported learning

Seventy-six student reaction evaluations alone or combined were conducted. In these evaluations, active learning was perceived to improve satisfaction in 66 studies (86.8%) and knowledge acquisition in 4 studies (5.3%). Sixty-five of these evaluations or studies compared active learning and lectures, 3 compared two active learning strategies, and 3 compared different forms of the same active learning strategy. In fifty-nine studies, active learning was perceived as superior to lectures, 5 found no differences between active learning and lectures, and only 1 reported lectures as superior to active learning. Only 4 evaluations reported instructors’ reaction data. In all these evaluations, instructors positively valued active learning.

Frequently, fairly, and occasionally evaluated (three or more studies) strategies using reaction-level data included flipped learning, PBL, group discussion, TBL, and blended learning. Peer teaching, role play debate, game, and think-pair-share were seldom evaluated (1 or 2 studies) using reaction data. Flipped learning was perceived to improve satisfaction in 16 studies and was regarded as superior to lectures in 16 studies. PBL was viewed as effective to improve knowledge acquisition in 2 studies and satisfaction in 13 studies and perceived as superior to lectures in 13 studies. Group discussion was deemed effective for knowledge acquisition in 1 study and satisfaction in 12 studies and reported to be superior to lectures in 12 studies. TBL was viewed as beneficial to improve knowledge in 1 study and satisfaction in 7 studies and considered more effective than lectures in 7 studies. Blended learning was deemed to improve satisfaction in 4 studies and regarded as superior to lectures in 4 studies.

### Learning-level evaluations

All studies in which learning was directly measured (n = 57) found that active learning was effective to improve knowledge acquisition largely based on test scores. Forty-eight of these studies (84.2%) compared active learning and lectures and 4 studies (7.0%) compared two active learning strategies. Based on the learning data, 39 studies found that active learning was superior to lectures in knowledge acquisition and 9 reported no difference between active learning and traditional lectures.

Frequently and fairly evaluated strategies using direct measures of learning included flipped learning, PBL, group discussion, and TBL. Blended learning, peer teaching, debate, game, and think-pair-share were rarely evaluated using such measures. Based on direct learning data, flipped learning was found to improve knowledge acquisition in 12 studies and to be more effective than lectures in knowledge acquisition in all 12 studies. Similarly, PBL was found to enhance knowledge acquisition in 9 studies and to be superior to traditional teaching in knowledge acquisition in all 9 studies. Direct learning data also supported the effectiveness of group discussion and TBL. Specifically, group discussion and TBL were found to improve knowledge acquisition in 5 and 7 studies, respectively. Regarding this outcome, group discussion was reported to be more effective than lectures in 5 studies and TBL in 7 studies.

## Discussion

Most studies on active learning in classroom dental education were quantitative in nature and published in the last decade, did not report the study methodology, performed outcome evaluations, engaged in post-intervention evaluations, relied on student data, mainly measured satisfaction and knowledge acquisition, and focused on clinical and basic sciences. Our review also revealed that flipped learning, group discussion, problem-based learning, and team-based learning were the active learning strategies most frequently evaluated in classroom dental education. Based on both reaction and factual (direct measure) data, these strategies improved satisfaction and knowledge acquisition and were superior to traditional lectures in improving these outcomes. To our knowledge, this is the first attempt to map the literature on active learning strategies in classroom dental education. Our findings provide a much-needed overview of this body of literature, which previous strategy-specific reviews were not in a position to provide [10, 16, 17]. Such an overview is of critical importance to describe the available evidence and inform future research directions on the study topic.

Consistent with the data from previous reviews, the number of studies on active learning in dental education has increased over time, especially within the last decade [9, 16, 18]. This shows a positive response to repeated calls for transforming the learning environments in dental education. This surge of publications is encouraging as a proxy for innovation in dental education and as a vehicle for knowledge dissemination among dental researchers and educators. In research, though, more publication does not necessarily mean better research activity. Although scoping reviews are not intended to assess the quality of the studies conducted and the credibility of the evidence generated [13], they can shed light on these issues based on the research methods and designs employed and the nature of the evidence produced. Quality of research in educational innovations can also be inferred by examining the types of evaluations conducted.

Most studies included in our review did not explicitly indicate the methodology used, which previous review research in medical education has also reported [19]. This is of concern as methodologies are supposed to be deliberately chosen to inform study designs [20]. We did not assess whether the reported methodologies were correctly classified; however, misclassifications of study methodologies have been documented [21, 22]. Such misclassifications may be due to lack of methodological understanding and attempts to pursue methodological credibility by claiming the use of “more robust” designs than those actually employed [22]. Several recommendations have been made to help researchers frame their projects methodologically and conceptually, including the engagement of methodologists throughout the research process [19].

Many studies included in our review employed a post-intervention evaluation design with a single cohort. This design is known to have several limitations, such as the inability to assess the magnitude of the improvement, if any, and to account for extraneous variables that may influence the learning outcomes apart from the intervention. Additionally, none of the studies included in our review reported process evaluation. This type of evaluation examines the extent to which an intervention was implemented as expected, met the parameters of effectiveness for the intervention (conditions under which it works), and was aligned with the underlying principles of the type of learning (e.g., collaborative learning) it aimed to promote [23]. Process evaluations are particularly helpful to determine whether an intervention did not work because of its effectiveness, implementation, or both. Failure to report process evaluation and properly design and implement active learning strategies have been previously documented [6]. Such shortcomings can be misleading in two fundamental ways: suggesting that a strategy was not effective when it could potentially be and suggesting it was delivered as expected when it was not.

Our findings highlight the importance of reporting not only the research inquiries (e.g., quantitative, qualitative) and methodologies (e.g., cross-sectional, RCT), but also the specific evaluation designs employed in the studies. Since methododologies may not be reported or properly classified, the specific evaluation design used becomes the best proxy for the quality of the outcome evaluation performed. This aspect should be determined by the researchers conducting the review because it may not be clearly defined in published papers. Our classification of evaluation designs can be used for this purpose, although further research may be needed to ascertain its instrumental value.

Few studies in our review used qualitative and mixed-method designs, which best practices in curriculum evaluation at the course and program levels recommend [24]. Such practices include using multiple evaluators, collecting and combining qualitative and quantitative data to provide a comprehensive evaluation, and using an evaluation framework (e.g., a logic model) to guide the evaluation process. Qualitative research is particularly suited to shed light into the circumstances under which interventions work (why and how) and the contextual factors shaping the outcomes of interventions and participants’ experiences [25].

Reviews on active learning in dental and medical education have revealed that active learning strategies are commonly evaluated using student feedback [6, 9]. Our study confirms the use of student feedback as the main source of evaluation, which is useful to judge some aspects of teaching effectiveness, such as engagement and organization, but not others such as appropriateness of the pedagogical strategy used to achieve the learning objectives [26]. Faculty feedback is important to comprehensively evaluate active learning across health professions education and ascertain their uptake and continued use of active learning strategies in classroom and clinical learning environments.

Similar to previous review research on active learning across health professions education [5], many studies included in our review used reactionary and factual data to evaluate the impact of several active learning strategies on the outcomes of interest, especially knowledge acquisition. This is an important strength of the literature on active learning in classroom dental education. Reaction- and learning-level outcome evaluations serve slightly different purposes, but both are needed to establish whether students and faculty are satisfied with the active learning strategies used and the actual impact of those strategies on knowledge acquisition, skill development, and attitudinal change. Further research is needed to critically appraise the validity of the means used to collect direct measures of learning, especially when knowledge tests were not originally developed and validated for research purposes.

Satisfaction and knowledge acquisition were the main outcomes evaluated in the studies included, while skill development (e.g., critical thinking, problem-solving skills) was infrequently considered. The latter is an important learning outcome in the context of competency-based education, which has been highly recommended in dental education [27]. Failure to measure whether active learning promotes important skills in this type of education may be due to the length and nature of the exposures (interventions) needed to achieve these outcomes and “inherent” difficulties to measure high-level outcomes [28].

Research on active learning in classroom dental education reflects the emphasis that traditional dental programs place on basic and clinical sciences. We identified only a few papers on active learning in behavioral and social sciences, which are a key component of dental education. These sciences have expanded the understanding of diseases beyond their biological determinants and that of treatment and management beyond clinical procedures [29]. Additionally, behavioral sciences provide dental students with competencies for personalized care, inter-professional care, disease prevention and management, and personal well-being of patients and care providers [30]. While integrating the behavioral science curriculum remains an important task [31], our findings suggest that research is warranted to demonstrate the effectiveness of active learning in delivering behavioral science content in dental education.

Active learning strategies most frequently evaluated in classroom dental education (flipped learning, group discussion, PBL, TBL) are similar to those commonly evaluated in dental and medical education [5, 9, 32]. Properly evaluated strategies provide dental educators with a menu of teaching options from which to choose the most suitable strategy(ies) to achieve their learning objectives. However, other active learning strategies (e.g., peer teaching, role play, think-pair-share) need to be further evaluated in dental education as they have proven effective to achieve certain learning objectives alone or in conjunction with other strategies [33, 34].

Despite the diversity of research designs, populations, settings, and evaluated strategies, active learning in classroom dental education was positively valued by students and faculty, was perceived as beneficial and ‘proven’ effective to promote satisfaction and knowledge acquisition, and was found to be superior to traditional lectures to promote these outcomes. These findings are consistent with those of previous reviews in dental education and health professions education in general [6, 9, 16]. Given the limitations of traditional lectures to promote deep and meaningful learning, dental researchers are encouraged to compare active learning strategies to achieve similar generic and specific learning objectives in order to demonstrate their relative effectiveness to achieve those objectives.

Our review also uncovered several reporting issues. These issues included not reporting or underreporting the research methodology, key aspects (e.g., allocation concealment) of the research design, characteristics of the instruments used for data collection, validity evidence of those instruments, active learning strategies employed, and length of the exposure to those strategies. Sufficient details of studies’ designs and conduct are important to judge the quality of the studies and that of the evidence produced. For example, without knowing the actual length of the exposure, it is not possible to appraise whether the expected learning outcomes were not achieved because the strategy used was not effective or because the exposure to the strategy was insufficient.

Limitations of our study encompasses general limitations of scoping reviews and study-specific limitations. General limitations include the potential for publication bias (published literature often prioritizes studies with significant findings over those with non-significant findings) and the absence of quality assessments for the included studies. While this assessment is not required in scoping reviews, it is important to note that the research designs of most included studies do not offer sufficient evidence to demonstrate the effectiveness of active learning in dental education classrooms. Several study-specific limitations need to be acknowledged. We relied on authors’ classifications of research methodologies and active learning strategies, which may not be the actual methods and strategies used. Misclassification of active learning strategies has been previously reported [17]. We excluded papers in languages other than English due to limited resources for translation, which may impact the generalizability of our study findings. However, based on the number of papers included, we are confident that the inclusion of this literature would not have changed the patterns observed in the extracted data. Our summary of the main results of previous studies by level of outcome evaluation (reaction and learning) may not account for noticeable differences in study design, sample, settings, and measures across studies.

## Conclusion

Although active learning strategies were positively valued and found effective using reaction and factual data, robust evaluation designs are needed to further demonstrate their effectiveness in classroom dental education. Aside from effectiveness questions, other issues remain to be elucidated, including for whom, how, when, and in what respect active learning may work in dental education. Future research should evaluate not only the impact of active learning strategies on satisfaction and knowledge acquisition, but also on skill development to support competency-based teaching and assessment in dental education. Similarly, active learning should be used and evaluated across all the main components of dental education, including behavioral and social sciences. Dental education journals should encourage researchers to comply with evaluation and reporting standards for educational innovations to ensure that these innovations are designed, conducted, and reported as expected.

## Supporting information

S1 Checklist Preferred Reporting Items for Systematic reviews and Meta-Analyses extension for Scoping Reviews (PRISMA-ScR) checklist. (DOCX)
