# Supplementary material for: Active learning in undergraduate classroom dental education- a scoping review
Source: PLoS One. 2023 Oct 26;18(10):e0293206. doi: 10.1371/journal.pone.0293206 (PMC10602256; doi:10.1371/journal.pone.0293206)
Supplement: Attachment — Submitted filename: Response to Reviewers.docx [file pone.0293206.s002.docx]

September 25, 2023

Dear Editor

Thank you very much for considering our manuscript titled “Active learning in undergraduate classroom dental education- a scoping review” (PONE-D-23-12023)

To the best of our ability, we addressed all the comments made by the editor and reviewers. Comments were pertinent and helpful.

Please see our point-by-point responses to the comments made by this reviewer.

Sincerely,

Arnaldo Perez, PhD

Assistant Clinical Professor

School of Dentistry

Faculty of Medicine & Dentistry

University of Alberta

**Author’s response to the Reviewers’ comments**

| **Comments** | **Author’s response** | **Location of the change** |
| --- | --- | --- |
| **Editor** | | |
| Highlight the limitations of the scoping reviews | Thank you so much for the feedback. We have included some general limitations of the scoping review. | Page 22. Last paragraph of the ‘Discussion’ section. |
| Highlight the limitations of the paper | We have included some additional limitations specific to our manuscript. | Page 22-23. Last paragraph of the ‘Discussion’ section. |
| Ensure that every recommendation is based on actual data in your review | We checked to the best of our ability to make sure that every recommendation is based on the actual data of our review. In the abstract, we replaced “Active learning should be encouraged” with “Active learning has the potential to improve student leanirng” | Conclusions in the abstract |
| **Reviewer 1** | | |
| Address any potential effect of not including non-English papers in the discussion section. | We have included this limitation and potential effect to our manuscript. | Page 22-23. Last paragraph of the ‘Discussion’ section. |
| **Reviewer 2** | | |
| Typos: Line 177: Methodologically | Sorry for the error. We have fixed the typo. | Page 13. First paragraph. |
